# Supplementary material for: Persuasive COVID-19 vaccination campaigns on Facebook and nationwide vaccination coverage in Ukraine, India, and Pakistan
Source: PLOS Glob Public Health. 2023 Sep 27;3(9):e0002357. doi: 10.1371/journal.pgph.0002357 (PMC10529538; doi:10.1371/journal.pgph.0002357)
Supplement: S1 Table — (DOCX) [file pgph.0002357.s001.docx]

**S1 Table. Ukrainian oblasts included in the study**

| 1 | Vinnytsia |
| --- | --- |
| 2 | Volyn |
| 3 | Dniprpetrovsk |
| 4 | Donetsk |
| 5 | Zhytomyr |
| 6 | Transcarpathian |
| 7 | Zaporozhye |
| 8 | Ivano-Frankivsk |
| 9 | Kirovograd |
| 10 | Luhansk |
| 11 | Lviv |
| 12 | Mykolaiv |
| 13 | Odesssa |
| 14 | Poltava |
| 15 | Rivne |
| 16 | Sumy |
| 17 | Ternopil |
| 18 | Kharkiv |
| 19 | Kherson |
| 20 | Khmelnytsky |
| 21 | Cherkasy |
| 22 | Chernivtsi |
| 23 | Chernihiv |
| 24 | Kyiv |
