# Supplementary material for: Persuasive COVID-19 vaccination campaigns on Facebook and nationwide vaccination coverage in Ukraine, India, and Pakistan
Source: PLOS Glob Public Health. 2023 Sep 27;3(9):e0002357. doi: 10.1371/journal.pgph.0002357 (PMC10529538; doi:10.1371/journal.pgph.0002357)
Supplement: S2 Table — (DOCX) [file pgph.0002357.s002.docx]

**S2 Table. Baseline COVID-19 vaccination coverage in Ukraine**

| **Oblasts** | **Baseline vaccination coverage second dose**  **Nov 8, 2021** | **Randomized Group** | **Population size**  **(Census 2020)** |
| --- | --- | --- | --- |
| **Vinnytsia** | 22.7% | 0 | 1538331 |
| **Volyn** | 17.0% | 0 | 1028693 |
| **Dnipropetrovsk** | 27.9% | 1 | 3173339 |
| **Donetsk** | 8.7% | 0 | 4118923 |
| **Zhytomyr** | 23.5% | 1 | 1208981 |
| **Transcarpathian** | 4.9% | 0 | 1250958 |
| **Zaporozhye** | 21.1% | 1 | 1686612 |
| **Ivano-Frankivsk** | 16.8% | 1 | 1365371 |
| **Kirovograd** | 20.6% | 0 | 926694 |
| **Luhansk** | 7.8% | 0 | 2131316 |
| **Lviv** | 23.1% | 0 | 2493714 |
| **Mykolaiv** | 23.6% | 1 | 1119147 |
| **Odessa** | 22.7% | 0 | 2366170 |
| **Poltava** | 30.7% | 0 | 1379140 |
| **Rivne** | 19.4% | 1 | 1151901 |
| **Sumy** | 25.8% | 0 | 1066055 |
| **Ternopil** | 19.9% | 0 | 1035444 |
| **Kharkiv** | 25.1% | 1 | 2642825 |
| **Kherson** | 25.0% | 1 | 1026481 |
| **Khmelnytsky** | 21.6% | 0 | 1251539 |
| **Cherkasy** | 26.6% | 1 | 1188508 |
| **Chernivtsi** | 18.9% | 1 | 898567 |
| **Chernihiv** | 24.5% | 1 | 982752 |
| **Kyiv** | 37.0% | 1 | 4701318 |
