# Supplementary material for: Persuasive COVID-19 vaccination campaigns on Facebook and nationwide vaccination coverage in Ukraine, India, and Pakistan
Source: PLOS Glob Public Health. 2023 Sep 27;3(9):e0002357. doi: 10.1371/journal.pgph.0002357 (PMC10529538; doi:10.1371/journal.pgph.0002357)
Supplement: S3 Table — (DOCX) [file pgph.0002357.s003.docx]

**S3 Table. Selected intervention districts five state trial through GeoLift in India**

| **District** | **State** | **District** | **State** |
| --- | --- | --- | --- |
| Aurangabad | Bihar | Balaghat | Madhya Pradesh |
| Begusarai | Bihar | Barwani | Madhya Pradesh |
| Bhojpur | Bihar | Betul | Madhya Pradesh |
| Buxar | Bihar | Bhind | Madhya Pradesh |
| Darbhanga | Bihar | Chhindwara | Madhya Pradesh |
| East Champaran | Bihar | Datia | Madhya Pradesh |
| Khagaria | Bihar | Hoshangabad | Madhya Pradesh |
| Madhepura | Bihar | Jhabua | Madhya Pradesh |
| Madhubani | Bihar | Katni | Madhya Pradesh |
| Munger | Bihar | Mandla | Madhya Pradesh |
| Muzaffarpur | Bihar | Mandsaur | Madhya Pradesh |
| Rohtas | Bihar | Morena | Madhya Pradesh |
| Samastipur | Bihar | Narsinghpur | Madhya Pradesh |
| Saran | Bihar | Rewa | Madhya Pradesh |
| Sitamarhi | Bihar | Sagar | Madhya Pradesh |
| Siwan | Bihar | Seoni | Madhya Pradesh |
| Vaishali | Bihar | Sidhi | Madhya Pradesh |
| Hazaribagh | Jharkhand | Singrauli | Madhya Pradesh |
| Jamtara | Jharkhand | Tikamgarh | Madhya Pradesh |
| Seraikela Kharsawan | Jharkhand | Vidisha | Madhya Pradesh |
