# Supplementary material for: Persuasive COVID-19 vaccination campaigns on Facebook and nationwide vaccination coverage in Ukraine, India, and Pakistan
Source: PLOS Glob Public Health. 2023 Sep 27;3(9):e0002357. doi: 10.1371/journal.pgph.0002357 (PMC10529538; doi:10.1371/journal.pgph.0002357)
Supplement: S4 Table — (DOCX) [file pgph.0002357.s004.docx]

**S4 Table. Districts in Pakistan included in the study**

|  | **District** |
| --- | --- |
| 1 | Abbottabad |
| 2 | Charsadda |
| 3 | Faisalabad |
| 4 | Gilgit |
| 5 | Gujranwala |
| 6 | Gujrat |
| 7 | Hyderabad |
| 8 | Islamabad |
| 9 | Karachi |
| 10 | Khairpur |
| 11 | Kotli |
| 12 | Lahore |
| 13 | Mardan |
| 14 | Mirpur |
| 15 | Multan |
| 16 | Muzaffarabad |
| 17 | Nowshera |
| 18 | Peshawar |
| 19 | Quetta |
| 20 | Rawalpindi |
| 21 | Sargodha |
| 22 | Sialkot |
| 23 | Skardu |
| 24 | Sukkur |
| 25 | Swat |
