# Supplementary material for: Persuasive COVID-19 vaccination campaigns on Facebook and nationwide vaccination coverage in Ukraine, India, and Pakistan
Source: PLOS Glob Public Health. 2023 Sep 27;3(9):e0002357. doi: 10.1371/journal.pgph.0002357 (PMC10529538; doi:10.1371/journal.pgph.0002357)
Supplement: S5 Table — (DOCX) [file pgph.0002357.s005.docx]

**S5 Table. Ad Metrics in Ukraine, India, and Pakistan**

|  | **Reach** | **Impressions** | **Clicks** | **Average Frequency** | **Cost per 1000 people reached** |
| --- | --- | --- | --- | --- | --- |
| **Ukraine** |  |  |  |  |  |
| Step 1  (12 oblasts) | 5,329,798 | 16,262,913 | 23,153 | 3.20 | 2.25 |
| Step 2  (24 oblasts) | 10,732,981 | 34,657,442 | 44,320 | 3.06 | 2.24 |
| Overall | 16,384,370 | 50,920,355 | 67,473 | 3.11 | 2.20 |
| **India** | | | | | |
| Hindi | 41,566,065 | 469,448,702 | 1,715,139 | 11.3 | 2.96 |
| English | 21,737,425 | 72,246,860 | 114,739 | 3.32 | 0.90 |
| Urdu | 9,109,491 | 15,917,812 | 111,751 | 1.75 | 0.47 |
| Overall | 42,811,254* | 557,613,374 | 1,941,629 | 13.02 | 3.43 |
| **Pakistan** | | | | | |
| Urdu | 23,944,008 | 123,290,025 | 500,774 | 5.15 | 1.10 |
| English | 28,330,593 | 128,784,303 | 398,332 | 4.55 | 0.93 |
| Overall | 52,274,601 | 252,074,328 | 899,106 | 4.82 | 1.01 |
| **This is unique reach overall – Facebook users could see ads in multiple languages.* | | | | | |
