# Supplementary material for: Persuasive COVID-19 vaccination campaigns on Facebook and nationwide vaccination coverage in Ukraine, India, and Pakistan
Source: PLOS Glob Public Health. 2023 Sep 27;3(9):e0002357. doi: 10.1371/journal.pgph.0002357 (PMC10529538; doi:10.1371/journal.pgph.0002357)
Supplement: S6 Table — (DOCX) [file pgph.0002357.s006.docx]

**S6 Table. Demographics of survey in Ukraine**

|  | **Survey 1**  **N=56,631** | **Survey 2**  **N=43,787** | **Survey 3**  **N=40,365** | **Overall**  **N=140,783** | **General population**  **Ukraine*** |
| --- | --- | --- | --- | --- | --- |
| **Gender** |  |  |  |  |  |
| Male | 8,258 (16%) | 6,234 (16%) | 5,154 (14%) | 19,736 (16%) | 46% |
| Female | 43,660 (84%) | 33,650 (84%) | 32,189 (86%) | 109,499 (85%) | 54% |
| **Age** |  |  |  |  |  |
| 18-29 | 7,116 (13%) | 5,116 (12%) | 7,546 (20%) | 19,778 (15%) | 13% |
| 30-44 | 18,684 (35%) | 13,689 (33%) | 14,385 (37%) | 46,758 (32%) | 24% |
| 45-59 | 18,158 (34%) | 14,117 (34%) | 10,928 (28%) | 43,203 (31%) | 21% |
| 60+ | 10,006 (19%) | 8,663 (21%) | 5,805 (15%) | 24,474 (18%) | 24% |
| **COVID-19 vaccination status** |  |  |  |  |  |
| Yes | 27,614 (63%) | 22,672 (69%) | 22,712 (73%) | 72,998 (68%) | 29% |
| No, but I have an appointment | 2,285 (5%) | 1,332 (4%) | 888 (3%) | 4,505 (4%) |  |
| No | 13,777 (32%) | 8,925 (27%) | 7,526 (24%) | 30,228 (28%) |  |
| Missing data:  Gender: 11,548 (8%), Age: 6,570 (5%), COVID-19 vaccination status: 33,052 (23%)  *Data on age and gender retrieved from general census in 2020, based on total population: <https://ukrstat.gov.ua/druk/publicat/kat_u/2021/zb/11/Yearbook_2020_e.pdf>  Vaccination coverage Ukraine in retrieved from: <https://ourworldindata.org/explorers/coronavirus-data-explorer?time=2021-12-04&facet=none&country=~UKR&Metric=People+vaccinated+%28by+dose%29&Interval=7-day+rolling+average&Relative+to+Population=true&Color+by+test+positivity=false>  COVID-19 vaccine acceptance in Dec 2020, before the roll out of the vaccination campaign: 61% would accept a COVID-19 vaccine, 16% would not and 23% did not know. Source: https://www.sciencedirect.com/science/article/pii/S0264410X22000998 | | | | | |
