# Supplementary material for: Persuasive COVID-19 vaccination campaigns on Facebook and nationwide vaccination coverage in Ukraine, India, and Pakistan
Source: PLOS Glob Public Health. 2023 Sep 27;3(9):e0002357. doi: 10.1371/journal.pgph.0002357 (PMC10529538; doi:10.1371/journal.pgph.0002357)
Supplement: S7 Table — (DOCX) [file pgph.0002357.s007.docx]

**S7 Table. Associations between the intervention and intentions to receive a COVID-19 vaccine among the unvaccinated in Ukraine**

|  | **Odds Ratio**  **95% CI** | **P-value** | **Adjusted Odds Ratio**  **95% CI** | **P-value** |
| --- | --- | --- | --- | --- |
| **Randomized group** |  |  |  |  |
| 5-weeks | Reference |  | Reference |  |
| 10-weeks | 0.83 (0.80-0.87) | 0.000 | 0.97 (0.88-1.07) | 0.531 |
| **Survey time** |  |  |  |  |
| Survey 1 | Reference |  | Reference |  |
| Survey 2 | 0.86 (0.82-0.91) | 0.000 | 0.89 (0.83-0.95) | 0.001 |
| Survey 3 | 0.80 (0.76-0.85) | 0.000 | 0.79 (0.71-0.88) | 0.000 |
| **Region** |  |  |  |  |
| West | Reference |  | Reference |  |
| Central | 0.88 (0.79-0.97) | 0.014 | 0.88 (0.79-0.98) | 0.018 |
| East | 1.07 (0.93-1.23) | 0.357 | 1.06 (0.92-1.22) | 0.420 |
| South | 0.84 (0.74-0.94) | 0.003 | 0.85 (0.76-0.96) | 0.008 |
| **Age** |  |  |  |  |
| 18-29 | Reference |  | Reference |  |
| 30-44 | 0.76 (0.72-0.82) | 0.000 | 0.77 (0.72-0.82) | 0.000 |
| 45-59 | 0.67 (0.63-0.72) | 0.000 | 0.67 (0.63-0.72) | 0.000 |
| 60+ | 0.67 (0.62-0.73) | 0.000 | 0.67 (0.63-0.73) | 0.000 |
| **Gender** |  |  |  |  |
| Male | Reference |  | Reference |  |
| Female | 1.58 (1.50-1.68) | 0.000 | 1.58 (1.49-1.67) | 0.000 |
| CI = Confidence Interval  *Adjusted for: all other variables in the table and oblast | | | | |
