# Supplementary material for: Persuasive COVID-19 vaccination campaigns on Facebook and nationwide vaccination coverage in Ukraine, India, and Pakistan
Source: PLOS Glob Public Health. 2023 Sep 27;3(9):e0002357. doi: 10.1371/journal.pgph.0002357 (PMC10529538; doi:10.1371/journal.pgph.0002357)
Supplement: S8 Table — (DOCX) [file pgph.0002357.s008.docx]

**S8 Table. Association between high trust in stakeholders and self-reported vaccination status in Ukraine**

|  | **Adjusted Odds Ratio**  **(95% Confidence Interval)** | **P-value** |
| --- | --- | --- |
| **Government** | 2.11 (1.83-2.42) | <0.001 |
| **Media** | 1.73 (1.50-2.01) | <0.001 |
| **Ministry of Health** | 3.17 (2.88-3.50) | <0.001 |
| **UNICEF** | 5.09 (4.71-5.51) | <0.001 |
| **Doctor** | 2.38 (2.25-2.51) | <0.001 |
| **Family/Friends** | 1.15 (1.10-1.20) | <0.001 |
