# Supplementary material for: Persuasive COVID-19 vaccination campaigns on Facebook and nationwide vaccination coverage in Ukraine, India, and Pakistan
Source: PLOS Glob Public Health. 2023 Sep 27;3(9):e0002357. doi: 10.1371/journal.pgph.0002357 (PMC10529538; doi:10.1371/journal.pgph.0002357)
Supplement: S9 Table — (DOCX) [file pgph.0002357.s009.docx]

**S9 Table. Association between low trust in stakeholders and self-reported vaccination status in Ukraine**

|  | **Adjusted Odds Ratio**  **(95% Confidence Interval)** | **P-value** |
| --- | --- | --- |
| **Government** | 0.21 (0.20-0.22) | <0.001 |
| **Media** | 0.25 (0.24-0.26) | <0.001 |
| **Ministry of Health** | 0.19 (0.18-0.20) | <0.001 |
| **UNICEF** | 0.22 (0.21-0.23) | <0.001 |
| **Doctor** | 0.32 (0.31-0.34) | <0.001 |
| **Family/Friends** | 0.47 (0.45-0.49) | <0.001 |
