# Supplementary material for: Persuasive COVID-19 vaccination campaigns on Facebook and nationwide vaccination coverage in Ukraine, India, and Pakistan
Source: PLOS Glob Public Health. 2023 Sep 27;3(9):e0002357. doi: 10.1371/journal.pgph.0002357 (PMC10529538; doi:10.1371/journal.pgph.0002357)
Supplement: S1 Text — (DOCX) [file pgph.0002357.s011.docx]

**S1 Text: Questionnaire for Ukraine**

| **Question** | **Answers** |
| --- | --- |
| How old are you? |  |
| What is your gender? | Male  Female  Not listed |
| Where do you live? | Dropdown list of 24 oblasts & Kyiv |
| **Vaccine Trust Indicator** | |
| If you are thinking about vaccination in general, can you say that you personally ... | Scale 1 -10 (0: strongly against vaccination, 5: I am neutral, 10: definitely for vaccination) |
| I generally trust vaccines or pharmaceutical manufacturers | 0: completely disagree, 5: neutral, 10: I completely agree |
| I generally trust the Ministry of Health | 0: completely disagree, 5: neutral, 10: I completely agree |
| I understand how vaccination helps my body fight infectious diseases | 0: completely disagree, 5: neutral, 10: I completely agree |
| I think it’s important to get vaccinated | 0: completely disagree, 5: neutral, 10: I completely agree |
| Vaccination is part of a healthy lifestyle | 0: completely disagree, 5: neutral, 10: I completely agree |
| **COVID-19 Vaccination status & intention** | |
| Have you received the COVID-19 vaccine? | - Yes - No, but I have an appointment - No |
| How likely are you to get the COVID-19 vaccine? | - Extremely likely - Somewhat likely - Neither likely nor unlikely - Somewhat unlikely - Extremely unlikely |
| You said yes, you got the COVID-19 vaccine. Have you received one or both doses of the COVID-19 vaccine | - I received both doses of a two dose vaccine - I received one dose of a two dose vaccine - I received one dose of one dose vaccine - I’m not sure |
| How likely is it that you will get a second dose of COVID-19 vaccine? | - Extremely likely - Somewhat likely - Neither likely nor unlikely - Somewhat unlikely - Extremely unlikely |
| **Vaccination beliefs among the vaccinated** | |
| How important do you think getting a COVID-19 vaccination was for your health? | - Extremely important - Very important - Moderately important - Slightly important - Not at all important |
| How much do you think getting a COVID-19 vaccine for yourself has protected other people in your family and community from COVID-19? | - A great deal - A lot - A moderate amount - A little - None at all |
| **Vaccination beliefs among the unvaccinated** | |
| How important do you think getting a COVID-19 vaccination is for your health? | - Extremely important - Very important - Moderately important - Slightly important - Not at all important |
| How much do you think getting a COVID-19 vaccine for yourself will protect other people in your family and community from COVID-19? | - A great deal - A lot - A moderate amount - A little - None at all |
| How safe do you think the COVID-19 vaccine is for you? | - Very safe - Mostly safe - Moderately safe - A little safe - Not at all safe |
| Do you know how to get vaccinated against COVID-19? For example, where you would go or how you would make an appointment? | - Yes - No - I’m not sure |
| What makes it hard for you to get a COVID-19 vaccine? | - I’m concerned about the long lines waiting for the vaccine - I’m concerned about the side effects and safety of the vaccine - It is hard for me to schedule a vaccine appointment date - I don’t trust those creating and distributing the vaccine - I don’t have transport to/from vaccination site or it’s too far away - I do not believe the COVID-19 vaccine works - I’m too busy to get the vaccine - I’m concerned that the vaccine was developed too quickly - I can’t (or can’t afford) to take time off of work - I plan to wait and see if it is safe, and maybe get it later - I don’t have adequate care for my own children while receiving the vaccine - I don’t trust the government - I don’t know how to schedule an appointment - I don’t think COVID-19 is a big enough risk for me to get this vaccine - I’ve already had COVID-19 and don’t feel I need a vaccine - Other, namely: |
| **All respondents** | |
| How worried are you about getting COVID-19? | - Extremely worried - Very worried - Somewhat worried - Slightly worried - Not worried at all |
| How worried are you about spreading COVID-19 to your friends and family? | - Extremely worried - Very worried - Somewhat worried - Slightly worried - Not worried at all |
| How much do you trust each of the following with the information that they provide about COVID-19 vaccination?   1. The government 2. The media/Press 3. The Ministry of Health 4. UNICEF 5. Your physician/family doctor 6. Your family/ friends | - None at all - A little - A moderate amount - A lot - A great deal |
| How much do you agree with this statement: “People should have the independence to make their own choice to get vaccinated against COVID-19”? | - Strongly agree - Somewhat agree - Neither agree nor disagree - Somewhat disagree - Strongly disagree |
| How likely are you to advise a close friend or relative to get vaccinated against COVID-19? | - Extremely likely - Somewhat likely - Neither likely nor unlikely - Somewhat unlikely - Extremely unlikely |
| When you think of most people whose opinion you value, how much would they approve of you getting a COVID-19 vaccine? | - A great deal - A lot - A moderate amount - A little - None at all |
| In your opinion, how many of the people whose opinion you value have received a COVID-19 vaccine? | - All of them - Most of them - About half of them - A few of them - None of them |
