# Supplementary material for: Persuasive COVID-19 vaccination campaigns on Facebook and nationwide vaccination coverage in Ukraine, India, and Pakistan
Source: PLOS Glob Public Health. 2023 Sep 27;3(9):e0002357. doi: 10.1371/journal.pgph.0002357 (PMC10529538; doi:10.1371/journal.pgph.0002357)
Supplement: Attachment — Submitted filename: Response letter PLOS GPH.docx [file pgph.0002357.s013.docx]

# Response letter

Dear editor and reviewers,

We would like to thank you for your reviews and your constructive comments. We have answered all points raised in the letter below; our answers are marked in blue.

Kind regards on behalf of all authors,

Maike Winters

## Editor comments

Dear Authors,

Thank you for submitting this article for consideration at PLOS Global Public Health. As you can see from the reviewer comments, both reviewers felt this was a strong paper but had what I felt were very useful suggestions to improve the framing and presentation of the article. I agree with all these comments. I have also attached some of my own feedback below. As a result, I am recommending a minor revision of the text. If the edits are made accordingly, I believe there is a good chance this article could be accepted at this journal.

Karen Grépin

Comments from Editor:

• How did you account for time in your randomized treatment? Those who received 10 weeks of treatment also had 5 more weeks of time to get vaccinated.

In the statistical analysis of the primary vaccination outcome in Ukraine, we accounted for time in multiple ways. First, we accounted for calendar time by including weekly categorical variables as predictors in the model. This allowed us to accommodate for any large-scale trends in vaccination that impacted all of Ukraine, outside of the treatment. Second, we included the oblast-level treatment status in the regression model as a time-varying predictor, where all regions began the study as untreated and then became treated at different calendar dates. Because our modeling was focused on monitoring the changes in vaccination across time, instead of more simply looking at the final study time period to determine if treated regions had larger increases in vaccination compared with the baseline rates, the impact of 5 vs. 10 weeks of treatment is directly accounted for in the analyses. This is a strength of the stepped wedge design that we utilized.

• In Ukraine, what was the timing of the 5 weeks of intervention in relation to the 10-week intervention? Was it at the start/finish?

The timing of the 5 weeks intervention was at the last (third) block of the stepped-wedge trial. The figure in the Supplementary Information (under Study Design Details) gives an overview of this. We have clarified this further in the manuscript by explicitly mentioning this figure, in lines 123-125:

*“At Step 2 all oblasts received intervention for the final five weeks, see Supporting Information for an overview of the study design.”*

• Ideally, you would have also had a control group that received no treatment to account for the additional time without any intervention. I think it needs to be made clearer that your intervention allows you to test the impact of 5 additional weeks of exposure to the intervention, not necessarily of the intervention itself.

The Ukraine study represents a stepped wedge randomized controlled trial, so that over time more oblasts received the treatment. It is a pragmatic study design that is an alternative to the traditional parallel cluster trial designs. In the first phase of the study (the first 5 weeks), none of the oblasts received the treatment. In the second phase, half of the oblasts received intervention, and in the third phase all oblasts received the intervention. In such a design, the distribution of results across unexposed observation periods is compared with that across the exposed observation periods. Thereby, the effect of the intervention itself can be determined.

Ref: <https://doi.org/10.1136/bmj.h391>

Also, the modeling of the data occurred on the weekly time scale, instead of at the final time point of the study (with a comparison to baseline rates). This means that at different study weeks, (i) none of the regions had been treated, (ii) only a subset of the regions had been treated, and (iii) all regions had been treated. This variation in treatment status over time, along with the focus of our analyses in modeling the trends in vaccinations across time, allows us to make inference on the different categories of treatment.

• Is it possible to provide balance tables to demonstrate how well the randomization was implemented? There is a risk due to the small number of geographic units that it was not.

We have added the table below with the baseline second dose vaccination coverage in the Supplementary Materials. At the start of the study (measured Nov 8, 2021), the average second dose vaccination coverage in the 5 week intervention group was: 19.6%. In the 10 week intervention group this was: 24.1%. We have further adjusted for baseline difference by including region as a random effect in our model. In the manuscript, we refer to the table in lines 125-129:

*“To determine which oblasts would receive the intervention for five weeks, we conducted a stratified randomization that balanced the intervention and control groups on observed COVID-19 vaccination coverage (from the week before the start of the study), population, and the most recently available estimates of the percentage of the population who lived in an urban setting, see Table S2.”*

| Oblasts | Baseline vaccination coverage  Second dose on Nov 8, 2021 | Randomized group  0= 5 weeks  1= 10 weeks | Population size (2020 census) |
| --- | --- | --- | --- |
| Vinnytsia region | 22.7% | 0 | 1538331 |
| Volyn region | 17.0% | 0 | 1028693 |
| Dnipropetrovsk region | 27.9% | 1 | 3173339 |
| Donetsk region | 8.7% | 0 | 4118923 |
| Zhytomyr region | 23.5% | 1 | 1208981 |
| Transcarpathian region | 14.9% | 0 | 1250958 |
| Zaporozhye region | 21.1% | 1 | 1686612 |
| Ivano-Frankivsk region | 16.8% | 1 | 1365371 |
| Kirovograd region | 20.6% | 0 | 926694 |
| Luhansk region | 7.8% | 0 | 2131316 |
| Lviv region | 23.1% | 0 | 2493714 |
| Mykolaiv region | 23.6% | 1 | 1119147 |
| Odessa region | 22.7% | 0 | 2366170 |
| Poltava | 30.7% | 0 | 1379140 |
| Rivne region | 19.4% | 1 | 1151901 |
| Sumy region | 25.8% | 0 | 1066055 |
| Ternopil region | 19.9% | 0 | 1035444 |
| Kharkiv region | 25.1% | 1 | 2642825 |
| Kherson region | 25.0% | 1 | 1026481 |
| Khmelnytsky region | 21.6% | 0 | 1251539 |
| Cherkasy region | 26.6% | 1 | 1188508 |
| Chernivtsi region | 18.9% | 1 | 898567 |
| Chernihiv region | 24.5% | 1 | 982752 |
| Kyiv region | 37.0% | 1 | 4701318 |

• I struggled to interpret table 2. Could these findings be presented in a different way?

The table has been replaced by the table below, which shows more clearly which associations were tested: between Vaccine Trust Indicator score and self-reported vaccination status, as well as between Vaccine Trust Indicator and self-reported vaccination intention. For both models, scoring low on the VTI was the reference group.

|  | **Vaccination status** | | **Vaccination intention** | |
| --- | --- | --- | --- | --- |
|  | **Adjusted OR (95% CI)** | **P-value** | **Adjusted OR (95% CI)** | **P-value** |
| **Vaccine Trust Indicator** |  |  |  |  |
| Low | Reference | - | Reference | - |
| Medium | 4.68 (4.49-4.87) | <0.001 | 6.47 (6.06-6.91) | <0.001 |
| High | 59.94 (55.14-62.99) | <0.001 | 29.98 (25.92-34.67) | <0.001 |
| OR = Odds Ratio, CI = Confidence Interval  Vaccination status: logistic regression. Vaccination intention: ordinal logistic regression  *adjusted for: randomized group, time (i.e., survey wave), region, age, gender, oblast | | | | |

• Why include Pakistan in this paper at all? The data are not randomized and really should be treated separately. Its outcomes are not even presented in the main tables. It feels like a vestige that might need dissection.

We decided to include Pakistan in this paper as it was part of the same campaign, which is why we felt the results should be presented here as well. While we couldn’t employ a randomized study design in Pakistan, we used a different, but commonly used quasi-experimental study design. The null results in Pakistan add to the overall findings and provide a holistic perspective of the campaign and its effects. We therefore would prefer to keep the Pakistan study in the paper.

• Why not also test whether the intervention affected vaccine intention using the randomization? This seems like missed opportunity.

We have tested this and described this in the manuscript, lines 444-448:

*“The intervention had no impact on the self-reported COVID-19 vaccination uptake among the survey respondents (aOR:0.98, 95% CI 0.93-1.04), see Table 1. The share of unvaccinated respondents who said it was unlikely that they would get vaccinated in the future increased slightly over time. There was no effect of the intervention on the likelihood that unvaccinated respondents would get vaccinated (aOR 0.97, 95% CI 0.88-1.07), table S3.”*

• I did not like this sentence: “This means that most people who 237 were eager to get vaccinated had already done so and those who initially opted to wait were likely also 238 vaccinated. This implies that the target audience may have been composed of people who were more 239 hesitant and less intentioned to get vaccinated – a more difficult audience to convince.” But in theory, that is exactly the audience the intervention aimed to convince, no?

Thank you for this comment. We approached this point in the discussion from the timing of our pilot study, where we tested which ads were most successful in each country. We believe that the target audience may have changed between the time of the pilot study (July - August 2021) and the time the three main studies were conducted (early 2022). We believe that the ‘moveable middle’ of people on the vaccine hesitancy continuum may have been easier to persuade than those who are on the extreme end of the continuum - who will likely be much more difficult to convince. We have softened the wording in the manuscript, lines 488-492:

*“Given the timing of our study in early 2022, many people who were eager to get vaccinated may have already done so, and those who initially opted to wait were likely to also have been vaccinated. This implies that the target audience may have shifted and been composed of people who were less intentioned to get vaccinated – a more difficult audience to convince.”*

## Reviewer 1

Reviewer #1: Summary:

This study has tried to assess, whether persuasive messages from UNICEF, disseminated via Facebook has any impact on the Covid 19 vaccine uptake in Ukraine, India and Pakistan. The study was designed as an RCT in Ukraine and India and in Pakistan it was deployed as a pre-post design with varying intervention periods in the three countries. The study concluded that there was no effect of intervention on vaccination uptake in all the three countries

General Remarks:

A well written piece of work. Authors have explored an interesting and important research area, which is very suitable in the current era, where internet and social media rules the world.

Thank you for these kind words.

Comments:

Methods section to be included after introduction and objectives and before results

We have changed the order of the manuscript following this comment - the Methods section now comes directly after the Introduction. The last two paragraphs of the Introduction have been removed, as in the previous version of the manuscript they aimed to briefly explain the methods before directly diving into the results. With the Methods section now directly following the Introduction, these paragraphs were no longer needed.

A small introduction about the study setting (especially about social media usage in the three countries) will help the international readers in understanding the social media use/influence in these countries

In Ukraine, we focused our efforts at a national level, including all oblasts as well as Kyiv city. The total Facebook user base of people aged 18-65+ years of age living in Ukraine at the time of the study was 22,310,200 - 24,810,200 users (roughly 60% of the adult population).

In India, our augmented synthetic control design generated 40 intervention districts across five States (Bihar, Madhya Pradesh, Jharkhand, Chhattisgarh, and Rajasthan). The total Facebook user base in these states was 81,900,000 users aged 18-65+ (roughly 36.5% of the adult population in those States).

In Pakistan, we focused our efforts on city districts prioritized for COVID-19 vaccination, and their catchment areas. The total Facebook user base of people aged 18-65+ years of age living in these districts was 32,938,300 (roughly 52% of those eligible for COVID-19 vaccines in those districts).

This has been added to the manuscript in the Methods section, under the subheading ‘Participants’ in lines 286-291:

*“The Facebook user base in Ukraine at the time of the study was between 22.3-24.8 million users, roughly 60% of the adult population. In India, there were approximately 81.9 million Facebook users in the 40 intervention districts, which corresponded to about 36% of the overall population in those states. Lastly, in Pakistan, the Facebook user base in the 25 city districts was around 32.9 million users, or around 52% of those eligible for COVID-19 vaccines in those districts.”*

Explain, why the intervention period and the study design are differing for the countries included in the study

For all three countries we assessed the feasibility of conducting an RCT. Being able to conduct an RCT required access to weekly COVID-19 vaccination data, a sufficient number of clusters to randomize for statistical power, and adequate Facebook usage among the population to target the campaigns on Facebook. In Ukraine, we had access to weekly COVID-19 vaccination data, but due to the relatively small number of oblasts, we opted for a stepped-wedge RCT, which would enhance the statistical power of the study.

In India as we had a large number of districts to randomize, we opted for a parallel arm RCT with an augmented synthetic control. Another consideration was the time available for the study. Conducting a stepped-wedge design requires a larger duration of time, which we did not have when the India study was undertaken.

In Pakistan, we opted for a pre-post design given the time constraints and preference of the stakeholders/government officials to message in all regions

In the manuscript, this has been updated in the lines 114-116:

*“For all three countries the feasibility to conduct an RCT was assessed. However, due to the availability and granularity of COVID-19 vaccination and the varying geographies, three different prospective study designs were employed for Ukraine, India, and Pakistan.”*

Lines 119-121:

*“In Ukraine, a stepped-wedge RCT was designed, with the 24 ‘oblasts’ (i.e., provinces, Kyiv region and Kyiv city were combined into one oblast) of the country as clusters. While the number of clusters was relatively small, the stepped-wedge design enhanced the statistical power of the trial.”*

Lines 192-194:

*“In India, the study was deployed in five states (Bihar, Chhattisgarh, Jharkhand, Madhya Pradesh, and Rajasthan). Due to the large number of districts in these states (174), we were able to use a parallel RCT, whereby 40 of the 174 districts were randomized to receive the intervention messages for six weeks.”*

Lines 205-208:

*“In Pakistan, due to limits in the availability of COVID-19 vaccination data, low statistical power and a preference of the stakeholders to message in all selected city districts, we opted for a pre-post design, whereby we followed 25 city districts during a period before the intervention, followed by a period of six weeks when all city districts received the intervention.”*

Specify, how informed consent was obtained from the participants for the online survey

Informed consent was obtained before the start of the survey. Potential participants were asked to read our information sheet online, containing information about the study as well as contact information for the Yale research team. Participants were then asked if they consented to this, and if agreed, they continued to the main survey. No names were recorded to enable the data to be collected anonymously. We have clarified this further in the manuscript, lines 216-219:

*“Informed consent was obtained before the start of the surveys in Ukraine. Potential participants were asked to read our information sheet online, containing information about the study as well as contact information for the Yale research team. Participants were then asked for their consent. Those who consented continued to the main survey.”*

## Reviewer 2

Reviewer #2: I have attached a file containing all of my comments to the authors.

Overall, I appreciate the endeavor on a large scale study and am eager to have it be added to the infodemic space. I have general concerns about the framing and scope of the study, and minor suggestions on improving the methods.

Thank you for an opportunity to review your work titled “Persuasive COVID-19 vaccination campaigns on Facebook and nationwide vaccination coverage in Ukraine, India, and Pakistan.” Also, thank you for your contributions to the infodemic space, specifically through exploring social media platforms as public health intervention platforms across three different settings collaborating across different sectors; a large endeavor.

Thank you for these kind words!

The paper assesses the effects of online messaging campaigns on Facebook on vaccination in three countries – Ukraine, India, and Pakistan.

The following are the comments I have, arranged by section. Briefly, overall, while the methods and results section reporting are strong, the piece can have better framing and tie-together in the introduction and conclusion. In addition, there are some concerns regarding the scope of conclusions drawn.

**Introduction**

In the second paragraph of the introduction, the ‘infodemic’ discussion on misinformation does not serve much purpose in the grand narrative of the paper. It serves as a transitionary paragraph into the harnessing of social media positively, but the space can flesh out this narrative instead. Specifically, why the choice of social media to implement an intervention study? By doing this, the authors can also better contextualise their findings in the grand scheme of infodemiology.

We have revised the second paragraph in the Introduction following this comment. It now reads in lines 61-65:

*“The ‘infodemic’ has caused an overload of (mis)information, making it difficult for the public to discern between trustworthy information and misinformation(5). Viral misinformation has not only undermined efforts of public health agencies and eroded trust, but also potentially increased vaccine hesitancy(6, 7). Social media platforms, with their global reach, can be a predominant source of vaccine misinformation and disinformation(8, 9).”*

From lines 100 – 102, a leap tries to link social media messaging to vaccine coverage, and linking the online to the offline. While the authors conducted the study well with the gold standard in epidemiological studies (RCTs, or RCT-style studies), the scope of infodemic studies is often different, and limited with conclusions it can draw on the causal front. This leap of logic to the causal is a deeper issue with infodemic studies: can they truly ever be causal? As the piece stands, the writing sounds more causal than the piece allows. To sort this issue out, I suggest some rewording or reframing to make the piece sound more associative than causal (this should also come out more in the conclusion).

Suggested response:

Thank you for raising this important point. Given the nature of randomized controlled trials, they are one of the strongest tools in a researcher’s toolbox regardless of the field of study as randomization in theory should minimize most of the confounding from measured as well as unmeasured (omitted) variables in the analysis making them better than other study designs. This can provide a causal interpretation to the results. However, conducting RCTs may not always be logistically possible. Our collaboration with the social media platform allowed us an opportunity to conduct such a study to test the effect of tailored social media messaging on intentions and actual vaccination coverage. As this is one of the initial studies of its kind, we have revised our manuscript by adding to the limitation, lines 564-565:

*“While two of the studies are RCTs, they are among the first in this field to use this type of study design. More RCTs in this field are needed to further establish causality.”*

**Methods**

It might be beneficial to compare the demographic information received from the surveys against those of Ukraine’s general census (assuming there is one). The authors can choose to put this into Table 1, or Table S1. Readers can see how the population recruited by Facebook might differ from the general population. For studies that recruit off social media platforms, there is an inherent bias in representativeness that reduces once better ‘triangulated’ by formally collected data. If there are any sort of regular vaccine trust / sentiment / hesitancy scores regularly or conducted once in Ukraine, this can also supplement Table 2.

We have added a column to Table S3 (in the previous version this was Table S3) with data from the general population from the 2020 census. In this extra column we similarly added the COVID-19 vaccination coverage in Ukraine at the start of the study (Nov 8, 2021). In the footnote of Table S3 we have also added COVID-19 vaccine acceptance in Ukraine, based on a survey carried out in December 2020 (<https://www.sciencedirect.com/science/article/pii/S0264410X22000998> ).

|  | **Survey 1**  **N=56,631** | **Survey 2**  **N=43,787** | **Survey 3**  **N=40,365** | **Overall**  **N=140,783** | **General population Ukraine*** |
| --- | --- | --- | --- | --- | --- |
| **Gender** |  |  |  |  |  |
| Male | 8,258 (16%) | 6,234 (16%) | 5,154 (14%) | 19,736 (16%) | 46% |
| Female | 43,660 (84%) | 33,650 (84%) | 32,189 (86%) | 109,499 (85%) | 54% |
| **Age** |  |  |  |  |  |
| 18-29 | 7,116 (13%) | 5,116 (12%) | 7,546 (20%) | 19,778 (15%) | 13% |
| 30-44 | 18,684 (35%) | 13,689 (33%) | 14,385 (37%) | 46,758 (32%) | 24% |
| 45-59 | 18,158 (34%) | 14,117 (34%) | 10,928 (28%) | 43,203 (31%) | 21% |
| 60+ | 10,006 (19%) | 8,663 (21%) | 5,805 (15%) | 24,474 (18%) | 24% |
| **COVID-19 vaccination status** |  |  |  |  |  |
| Yes | 27,614 (63%) | 22,672 (69%) | 22,712 (73%) | 72,998 (68%) | 29% |
| No, but I have an appointment | 2,285 (5%) | 1,332 (4%) | 888 (3%) | 4,505 (4%) |  |
| No | 13,777 (32%) | 8,925 (27%) | 7,526 (24%) | 30,228 (28%) |  |
| Missing data:  Gender: 11,548 (8%), Age: 6,570 (5%), COVID-19 vaccination status: 33,052 (23%)  *Data on age and gender retrieved from general census in 2020, based on total population:<https://ukrstat.gov.ua/druk/publicat/kat_u/2021/zb/11/Yearbook_2020_e.pdf>  Vaccination coverage Ukraine in retrieved from:<https://ourworldindata.org/explorers/coronavirus-data-explorer?time=2021-12-04&facet=none&country=~UKR&Metric=People+vaccinated+%28by+dose%29&Interval=7-day+rolling+average&Relative+to+Population=true&Color+by+test+positivity=false>  COVID-19 vaccine acceptance in Dec 2020, before the roll out of the vaccination campaign: 61% would accept a COVID-19 vaccine, 16% would not and 23% did not know. Source: https://www.sciencedirect.com/science/article/pii/S0264410X22000998 | | | | | |

There’s little elaboration on why only Ukraine has a survey implemented, and why the different countries have different study designs. I gather that this is due to very deep issues in planning and coordination. This amendment can be in lines 315-316; or, an elaboration when the authors explicate on each country.

We had conducted similar surveys in India and Pakistan, but the sample sizes were either too small (in India) or homogenous (79% men in Pakistan, 71% in India) to draw conclusions and thus excluded from the manuscript. Further, for those who did manage to get through and finish the survey (in India we had a total of 1,009 people who had data on self-reported COVID-19 vaccination status, in Pakistan 4,466), the data were highly skewed, with overall self-reported vaccination rates of 97% in both countries. Given the low yield and the highly skewed results, we decided not to include the survey data for India and Pakistan in this study.

In the supplementary files, there is no detail on the 25 districts studied in Pakistan

Thank you for noticing this. We have now added a table in the Supplementary files with an overview of the 25 districts in Pakistan.

|  | **District** |
| --- | --- |
| 1 | Abbottabad |
| 2 | Charsadda |
| 3 | Faisalabad |
| 4 | Gilgit |
| 5 | Gujranwala |
| 6 | Gujrat |
| 7 | Hyderabad |
| 8 | Islamabad |
| 9 | Karachi |
| 10 | Khairpur |
| 11 | Kotli |
| 12 | Lahore |
| 13 | Mardan |
| 14 | Mirpur |
| 15 | Multan |
| 16 | Muzaffarabad |
| 17 | Nowshera |
| 18 | Peshawar |
| 19 | Quetta |
| 20 | Rawalpindi |
| 21 | Sargodha |
| 22 | Sialkot |
| 23 | Skardu |
| 24 | Sukkur |
| 25 | Swat |

In line 369 under “Intervention”, the sentence reads “The control condition received no messages” – I believe this is untrue for Ukraine; the control condition received only 5 weeks compared to the 10.

We have added a sentence to further explain how the control condition worked in Ukraine. Lines 225-227:

*“The control condition received no messages. In Ukraine, the treatment status of each region changed over time, with all regions beginning in the control condition.”*

**Results**

Make sure the table headings (line 201, Table 1; line 212, Table 2; Table S3, S4, S5 supplementary) include the country so that interpretation is clear. I can make this out generally based on what the authors researched in each country, but it would help the reader.

We have added the country to all table headings.

Table S1: Putting the asterisk for the footnote of ‘India, Overall’ onto the ‘Overall’ part, for both India and Pakistan, assuming that the calculation is the same, because both have multiple languages.

The asterisk in Table S1 refers only to ‘India, Overall’, because in India, Facebook users could see ads in multiple languages. In Pakistan on the other hand, the various languages were set up as separate campaigns which precluded users from being able to see them in multiple languages. We have clarified this in the footnotes of Table S1:

*“*This is unique reach overall – Facebook users in India could see ads in multiple languages, while those in Pakistan were set-up as separate campaigns.”*

**Discussion**

In line 234, the timing of interventions can also be a strength to the piece. In essence, any associated upward trend is actually because of the campaign, not because there is some underlying, unmet demand.

Thank you for pointing this out. We agree that in theory the timing could be a strength of the study. The editor commented on this section of the discussion as well. We have incorporated both comments in the revision of the paragraph, lines 488-494:

*“Given the timing of our study in early 2022, many people who were eager to get vaccinated may have already done so, and those who initially opted to wait were likely to also have been vaccinated. This implies that the target audience may have shifted and been composed of people who were less intentioned to get vaccinated – a more difficult audience to convince. On the other hand, the timing of the intervention may have meant that there was to a large extent no unmet demand, which would imply that any changes we observed in vaccination coverage would be linked to the intervention.”*

Lines 256-260 about the external events needs more elaboration. Behaviour is a complex outcome from various external forces and internal considerations, and the authors should caveat the piece with this as a central point. This point corresponds to what I wrote in the ‘introduction’ comments. This can either be included in the discussion section or the strengths and limitations.

We have added the following to this paragraph, lines 524-527:

*“Behavior is inherently complex and is influenced by various factors, from internal considerations to socio-economic circumstances (Ref: Behavioural Drivers model UNICEF) and previously described external factors. Interventions such as ours likely play a small role in the decision making process of vaccination practices.”*
